# Tetracycline Adsorption Performance and Mechanism Using Calcium Hydroxide-Modified Biochars

**DOI:** 10.3390/toxics11100841

**Published:** 2023-10-07

**Authors:** Kaifeng Wang, Runlin Yao, Dongqing Zhang, Na Peng, Ping Zhao, Yongming Zhong, Haijun Zhou, Jiahui Huang, Chen Liu

**Affiliations:** 1 Guangdong Provincial Key Laboratory of Petrochemical Pollution Process and Control, Key Laboratory of Petrochemical Pollution Control of Guangdong Higher Education Institutes, School of Environmental Science and Engineering, Guangdong University of Petrochemical Technology, Maoming 525000, China; kfwang11@163.com (K.W.); dqzhang3377@outlook.com (D.Z.); zhongym@gdupt.edu.cn (Y.Z.); zhouhaijun_2000@126.com (H.Z.); hjh960409@163.com (J.H.); lc1272739015@163.com (C.L.); 2 Bathurst Future Agri-Tech Institute, Qingdao Agricultural University, Qingdao 266109, China; qrrunlinyao0430@163.com; 3 Geological Party 105, Guizhou Provincial Bureau of Geology and Mineral Exploration and Development, Guiyang 550018, China; 13885102199@126.com

**Keywords:** biochar, calcium hydroxide, agricultural and forestry wastes, tetracycline, adsorption

## Abstract

Tetracycline is frequently found in various environments and poses significant ecological risks. Calcium hydroxide-modified biochar has shown potential as a material for removing multiple classes of pollutants from wastewater streams. The tetracycline-adsorption performance and mechanism of alkali-modified biochars derived from nine wastes (corn straw, rice straw, swine manure, cypress powder, wheat straw, peanut shell, walnut shell powder, soybean straw, and corncobs) were investigated in the study. Among the four alkalis tested, calcium hydroxide exhibited the most effective modification effects at a pyrolysis temperature of 500 °C. Straw biomass was most suitable to be modified by calcium hydroxide, and calcium hydroxide-modified biochar showed the highest adsorption performance for tetracycline. The maximum adsorption capacities were 8.22 mg g^−1^ for pristine corn straw biochar and 93.46 mg g^−1^ for calcium hydroxide-modified corn straw biochar. The tetracycline adsorption mechanism by calcium hydroxide-modified corn straw biochar involved hydrogen bonding, oxygen-containing functional groups, Ca^2+^ metal complexation, and electrostatic attraction. Consequently, calcium hydroxide-modified corn straw biochar emerges as an environment-friendly, cost-effective, and efficient tetracycline adsorbent.

## 1. Introduction

Tetracycline (TC) is a commonly used antibiotic, and its extensive misuse and poor absorption and metabolism in humans and animals have led to their frequent detection in aquatic environments [1,2]. Even at low concentrations between ng L^−1^ and μg L^−1^, TC can cause direct toxicity to aquatic organisms and harm antibiotic-resistance genes, posing significant ecological risks [3,4].

TC is frequently found in various environments [5,6]. Several physicochemical technologies have, thus, been explored for TC removal, including advanced oxidation [7] and photolysis [8]. These techniques are often associated with high cost, energy consumption, and toxicity. In contrast, adsorption is an appealing approach, particularly for large-scale applications, because of its operational flexibility, cost-effectiveness, and ease of reversibility [9,10].

Utilising environment-friendly adsorbents is a promising strategy for immobilising antibiotics in wastewater streams. Biochars derived from manure, agricultural byproducts, and food waste have gained attention as a potential material for removing various classes of pollutants from wastewater streams [9,11]. However, most pristine biochars exhibit poor TC adsorption capacity. For example, the TC adsorption capacities of biochar derived from waste dregs (*Auricularia auricular*) and rice straw were 9.90 mg g^−1^ [12] and 14.19 mg g^−1^ [13], respectively. Therefore, exploring novel methods that can improve the graphitisation degree, optimise surface structure, and enrich the types of surface functional groups for modified biochar preparations are of great significance for optimal TC capture [14,15].

Strong alkaline reagents such as sodium hydroxide and potassium hydroxide have shown promising modification effects. For example, the maximum TC adsorption capacity of potassium hydroxide-modified biochars derived from rape straw was 325.07 mg g^−1^ [16], whereas that of sodium hydroxide-modified biochar derived from waste coffee grounds was 113.64 mg g^−1^ [17]. The modification mechanism of biochar using strong bases involves changes in specific surface areas, pore structure, functional groups, and surface charge [16,18]. However, despite the fact that strong alkali-modified biochars substantially improve the TC adsorption capacity, challenges associated with the preparation process remain. The use of strong alkalis in the modification process can lead to biomass decomposition, necessitating a complex two-step pyrolysis process that involves the pyrolysis of biomass into biochar and the subsequent pyrolysis of mixed alkali and biochar [18,19]. Furthermore, because the first and second pyrolysis steps require 1–2 h at temperatures ≥ 500 °C and 1–3 h at temperatures ≥ 600 °C, respectively, energy consumption and costs are typically high [20]. In addition, the preparation process may lead to secondary pollution, such as the release of a large amount of Na^+^ into the environment and the formation of saline-alkali soil, thereby threatening ecosystems [21].

Currently, the modification of biochar using calcium hydroxide has gained attention as it allows for direct mixing with biomass during pyrolysis at low temperatures to produce biochar [22,23,24,25]. Nevertheless, calcium hydroxide-modified biochar is primarily employed to recover phosphorus from environmental matrices, and a limited number of reports are available on its use for contaminant remediation purposes [22,23]. Utilising agricultural and forestry wastes as biomass to remove contaminants offers an environment-friendly approach [10,26]. Various agricultural wastes, including rice straw, corn straw, wheat straw, coconut, rice husks, coffee grounds, and sawdust, have been successfully used to remove heavy metal ions and antibiotics because of their accessibility, low cost, and high adsorption capacity [9,10].

The TC adsorption performance and mechanism of four alkali-modified biochars derived from nine wastes were investigated in the study, exploring influencing factors and underlying adsorption mechanisms. We examined several aspects influencing TC adsorption performance, including pyrolysis temperature, biomass type, chemical reagent type, and calcium hydroxide loading rate. Moreover, we elucidated the mechanisms responsible for TC adsorption by calcium hydroxide-modified biochars through biochar characterisation. The findings of this study contribute to the efficient treatment of wastewater-containing antibiotics.

## 2. Materials and Methods

### 2.1. Biomass and Chemicals

Nine different raw materials, including rice straw (RS), swine manure (SM), corn straw (CS), cypress powder (CP), wheat straw (WS), peanut shell (PS), walnut shell powder (WP), soybean straw (SS), and corncobs (CC), were utilised to prepare the biochar. Swine manure and rice straw were sourced from Hunan Province, cypress powder was obtained from Heilongjiang Province, and the remaining six raw materials were collected from farmlands in Henan Province, China. Analytical-grade TC (>96 wt%) and other chemical reagents were procured from Shanghai Aladdin Chemistry Co., Ltd., Shanghai, China.

### 2.2. Biochar Preparation

The biochar was prepared according to a previous study [25]. Briefly, the preparation of pristine and modified biochar involved pyrolysis in a corundum crucible in an oxygen-limited environment (without using nitrogen). The pyrolysis process via a muffle furnace was conducted at 400–600 °C for 2 h with a heating rate of 20 °C min^−1^. The modified biochar was prepared by mixing the biomass with solutions or suspensions containing 10, 20, and 30% (*w*/*w*) of each of four alkalis (calcium hydroxide, magnesium hydroxide, sodium hydroxide, or potassium hydroxide). The pristine biochar and modified biochar were naturally cooled and used for the subsequent adsorption experiments.

### 2.3. Biochar Characterisation

The pH of the biochar samples was measured using a Rex PHS-3C digital pH metre (INASE Scientific Instrument Co., Ltd., Shanghai, China). The specific surface areas and pore structures of the samples were determined using a Micromeritics ASAP-2460 instrument (Micromeritics, GA, USA). The levels of organic elements, including carbon (C), nitrogen (N), hydrogen (H), and oxygen (O), were analysed using a CHNS/O Analyser (Elementar Analysensysteme GmbH, Hanau, Germany). The structural morphology changes were observed using a Hitachi S-3400N scanning electron microscope (SEM) (Hitachi Ltd., Tokyo, Japan). The surface elemental composition was measured using energy-dispersive X-ray spectroscopy (EDX; IXRF Systems, Austin, TX, USA). The surface functional groups and crystal structures were analysed using a Nicolet iS10 spectrometer (Thermo Fischer Scientific, Waltham, MA, USA) and a Bruker D8 X-ray diffractometer (XRD; Bruker, Berlin, Germany), respectively. Fourier transform infrared (FTIR, Thermo Fisher, Waltham, MA, USA) spectroscopy was performed using a Nicolet iS10 spectrometer; FTIR used the transmission for sample analysis, and the samples were diluted with KBr before instrumental analysis.

### 2.4. Adsorption Experiments

The adsorption tests were conducted in 250-mL conical flasks containing 100 mL of TC solutions and 0.1 g of biochar. Initially, the TC was dissolved in 2 mL of methanol, a commonly used mobile phase in liquid chromatography, to enhance dissolution and prevent TC precipitation. The concentration of the TC stock solution is 1000 mg L^−1^, and it should be stored in the dark. Then, the TC stock solution was diluted to 100 mL with ultrapure water. The mixture of biochar and TC solution was shaken at 180 rpm for 6 h.

Under the same experimental conditions, 0.1 g of either pristine or modified biochar was added to flasks containing 100 mL of 50 mg L^−1^ TC. The flasks were shaken at 180 rpm for 6 h. The effects of the alkali reagent type, raw materials, pyrolysis temperature, and amount of alkali added on TC adsorption capacity were assessed to identify the modified biochars with the highest TC adsorption capacity.

To investigate TC adsorption kinetics, 0.1 g of corn straw biochar (CSB) or calcium hydroxide-modified CSB (Ca-CSB) was added to flasks with 100 mL of TC at different concentrations (10 mg L^−1^ for CSB and 50 mg L^−1^ for Ca-CSB). The biochar and TC solution mixtures were centrifuged at 180 rpm, and the contact time required for adsorption equilibrium was set at 1440 min. Samples were collected after 1, 2, 3, 4, 5, 10, 15, 30, 60, 90, 150, 210, 270, 350, 500, 720, and 1440 min. The kinetic data were fitted using four dynamic models: pseudo-first-order (PFO), pseudo-second-order (PSO), intra-particle diffusion (IPD), and Evolich models (Table 1).

For the TC adsorption isotherms, 0.1 g of CSB or Ca-CSB was added to flasks containing 100 mL of TC at different concentrations (2.5–70 mg L^−1^ for CSB and 10–120 mg L^−1^ for Ca-CSB). The flasks were shaken in an oscillator at 25 °C for 24 h at a constant speed of 180 rpm. The isotherm data were fitted to the Langmuir and Freundlich models (Table 1).

To investigate the impact of pH on adsorption performance, 0.1 g of Ca-CSB was added to flasks containing 100 mL of 50 mg L^−1^ TC. The flasks were shaken at 180 rpm for 6 h. The pH range was set from 2 to 12. The experiment was divided into two groups: (i) adjusting the initial pH of the TC solution with 0.1 M NaOH or 0.1 M HCl before adding biochar and (ii) adjusting the equilibrium pH with a mixture of TC solution and 0.1 g of biochar using 0.1 M NaOH or 0.1 M HCl.

For TC concentration determination, the supernatant was filtered using a 0.22-μm nylon fibre filter after sampling and injected into 2-mL high-performance liquid chromatography (HPLC) bottles. The TC concentration in the filtrate was measured using an HPLC Essentia LC-16 (SHIMADZU, Kyoto, Japan) with a C-18 column (Phenomenex Inc., Torrance, CA, USA) and a UV–vis detector. The mobile phase consisted of water with 0.1% formic acid and methanol (65:35, *v*/*v*). The column temperature was 35 °C, the flow rate was 0.8 mL min^−1^, and the retention time was 10 min.

## 3. Results and Discussion

### 3.1. Pristine and Modified Biochar Adsorbent

**Figure 1:**
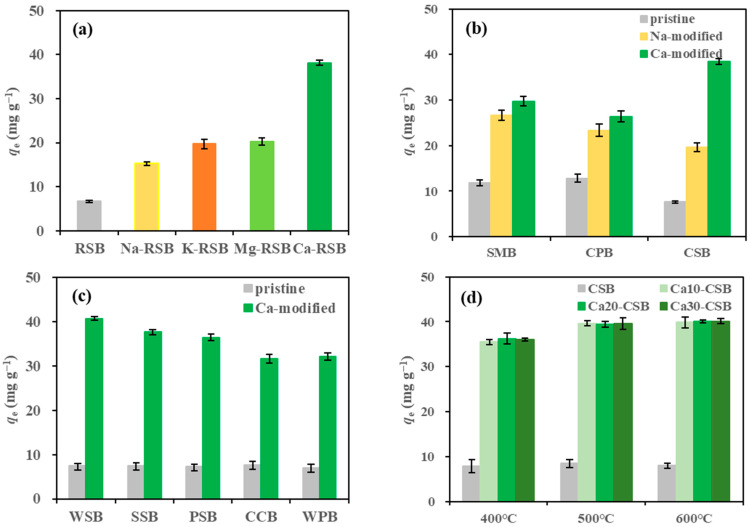
Effects of (**a**) alkali type, (**b**) Ca vs. Na modification, (**c**) biomass type, and (**d**) pyrolysis temperature and amount of calcium hydroxide added on the tetracycline (TC) adsorption performance using the pristine and modified biochar derived from different raw materials. RSB: rice straw biochar, SMB: swine manure biochar, CPB: cypress powder biochar, CSB: corn straw biochar, WSB: wheat straw biochar, SSB: soybean straw biochar, PSB: peanut shell biochar, CCB: corncob biochar, WPB: walnut shell powder biochar (biochar dosage: 1.0 g L^−1^; TC concentration: 50 mg L^−1^; initial TC solution pH: 6.0; shaking time: 6 h; 25 °C).

In total, 31 biochars were prepared for TC adsorption, as shown in Figure 1a–d. The TC adsorption capacity of the four alkali-modified rice straw biochars (RSB; 15.28–38.12 mg g^−1^) was significantly higher than that of the pristine RSB (6.71 mg g^−1^; Figure 1a). Differences between the four alkalis were observed in their modification effects on TC adsorption capacity. The order of the four alkalis from the highest to lowest level of modification was calcium hydroxide, magnesium hydroxide, potassium hydroxide, and sodium hydroxide. This may have been due to the strong effect of sodium hydroxide and potassium hydroxide as catalysts on the structure of biomass, causing the collapse and aggregation of the pore structure of the modified biochar, reducing specific surface area and porosity [27,28]. Therefore, the TC adsorption performance of the modified RSB was closely related to the type of alkali.

To explore the effect of biomass type on TC adsorption, representative agricultural wastes were selected, including swine manure, cypress powder, and corn straw (Figure 1b). The adsorption capacity of cypress powder biochar (12.82 mg g^−1^) was slightly higher than that of swine manure biochar (11.80 mg g^−1^), both of which were higher than that of CSB (7.61 mg g^−1^). Compared with those of pristine biochars, the adsorption capacities of the three biochars modified with sodium hydroxide increased 1.26, 0.82, and 1.57 times, respectively; in contrast, the adsorption capacities of the three calcium hydroxide-modified biochars increased by 1.52, 1.06, and 4.05 times, respectively. This indicates that calcium hydroxide-modified biochars exhibited superior activation effects on the three types of biochars, particularly CSB.

The composition of the biochar feedstock (wheat straw, soybean straw, peanut shell, walnut shell powder, and corncob) influenced biochar TC adsorption (Figure 1c). The TC adsorption capacities of the pristine biochars derived from these five waste types ranged from 6.95–7.65 mg g^−1^. Among the selected agricultural and forestry wastes, corncob biochar exhibited the highest TC adsorption capacity (7.65 mg g^−1^), whereas after calcium hydroxide modification, it showed the lowest TC adsorption capacity (31.63 mg g^−1^). Nevertheless, even after modification, corncob biochar exhibited a significantly higher TC adsorption capacity than swine manure and cypress powder biochars. This finding suggests that calcium hydroxide is suitable for modifying biochar derived from plant straw biomass. This may be attributed to the inability to fully ionise with calcium hydroxide in water, a relatively weak alkalinity, and the fact that plant straw containing more cellulose with a relatively simple structure makes it easier to decompose than lignin and other components with more complex structures. Pyrolysis temperature also plays a crucial role in TC adsorption performance. To assess the effects of pyrolysis temperature and the addition rate of calcium hydroxide on TC adsorption capacity, corn straw, which is locally abundant, was selected as the representative raw material for biochar. As presented in Figure 1d, the adsorption performance of pristine CSB at temperatures between 400–600 °C was similar, with an adsorption capacity of approximately 8 mg g^−1^. Adding calcium hydroxide significantly increased the TC adsorption capacity of CSB. At temperatures of 400, 500, and 600 °C, the adsorption capacity of CSB prepared with 10% calcium hydroxide was 3.49, 3.69, and 4.01 times higher than that of pristine CSB, respectively. Higher pyrolysis temperatures positively correlated with the modification effect of calcium hydroxide within the temperature range of 400–600 °C. The difference in TC adsorption performance among the different pyrolysis temperatures was insignificant. At the same pyrolysis temperature, different addition rates of calcium hydroxide had an insignificant effect on CSB adsorption capacity. For example, at 500 °C, adding 10, 20, and 30% of calcium hydroxide increased the TC adsorption capacity of CSB approximately 3.68 times. Considering the economic cost and adsorption capacity, CSB with 10% calcium hydroxide pyrolysed at 500 °C was selected for subsequent research.

### 3.2. Influence of Solution pH on TC Adsorption

**Figure 2:**
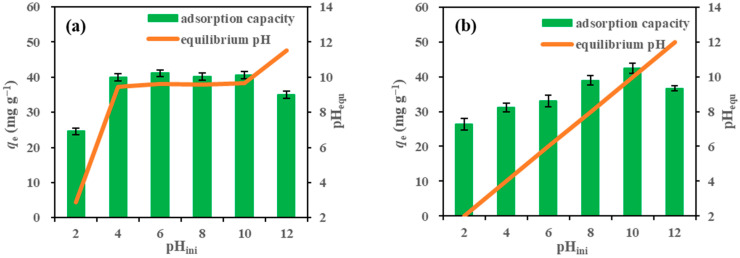
Adsorption capacity of tetracycline (TC) (**a**) by calcium hydroxide-modified corn straw biochar (Ca-CSB) at different initial pH solutions and (**b**) by Ca-CSB at different equilibrium pH solutions (biochar dosage: 1.0 g L^−1^; TC concentration: 50 mg L^−1^; shaking time: 6 h; 25 °C).

The initial TC adsorption capacity of Ca-CSB was investigated over a range of initial solution pH values, as shown in Figure 2a. When the initial solution pH was adjusted to 4.0–10.0, the solution pH after reaching adsorption equilibrium was approximately 9.6, indicating that Ca-CSB exhibited strong alkalinity and buffering properties [16]. The adsorption capacity of Ca-CSB at pH = 4.0 (39.95 mg g^−1^) was significantly higher than that at pH = 2.0 (24.58 mg g^−1^). It remained constant at pH 4.0–10.0 (39.95–41.13 mg g^−1^) and then decreased at pH = 12.0 (35.06 mg g^−1^). Therefore, the optimal pH for TC adsorption by Ca-CSB was 9.6. This observation that TC adsorption remained constant in the pH range of 4.0–10.0 is consistent with the previous observations [29].

Solution pH_ini_ refers to adjusting the pH to 2.0, 4.0, 6.0, 8.0, 10.0, and 12.0 after adding Ca-CSB (Figure 2b). As shown in Figure 2b, the TC adsorption capacity of Ca-CSB increased with an increase in pH within the pH_ini_ range of 2.0–10.0 and decreased at pH = 12.0. The highest TC adsorption capacity of Ca-CSB (42.37 mg g^−1^) was observed at pH = 10.0. The buffering properties of Ca-CSB allowed it to maintain efficient TC adsorption performance over a wider pH range from 4.0–10.0.

The optimal TC adsorption capacity of biochar is influenced by the existing form of TC at different pH conditions [23] and the pH at the point of zero charge (pH_pzc_) of the biochar [29]. TC primarily exists in a cationic form (TC^+^) at pH < 3.3, a neutral form (TC^0^) at pH = 3.4–7.7, an anionic form (TC^−^) at pH = 7.6–9.7, and another anionic form (TC^2−^) at pH > 9.7.

In this study, the pH_pzc_ of Ca-CSB was determined to be 9.8. At pH 2.0, the surface of Ca-CSB exhibited a large positive charge. Due to the electrostatic repulsion between Ca-CSB and TC^+^, the adsorption of TC was the lowest (26.32 mg g^−1^). At pH = 4.0–10.0, the number of positive charges on the surface of Ca-CSB gradually decreased, and the form of TC changed from TC^0^ to TC^−^, indicating that electrostatic attraction was not the primary driving force for TC adsorption. This suggests the involvement of other mechanisms in the TC adsorption process, such as π–π binding, hydrogen bonding, and hydrophobic interactions. At pH = 12.0, the surface of Ca-CSB became negatively charged, resulting in a significantly lower TC adsorption capacity (36.63 mg g^−1^) compared to that at pH = 10.0 (42.37 mg g^−1^) due to strong repulsive forces between Ca-CSB and TC^2−^.

### 3.3. Adsorption Kinetics and Isotherms

**Figure 3:**
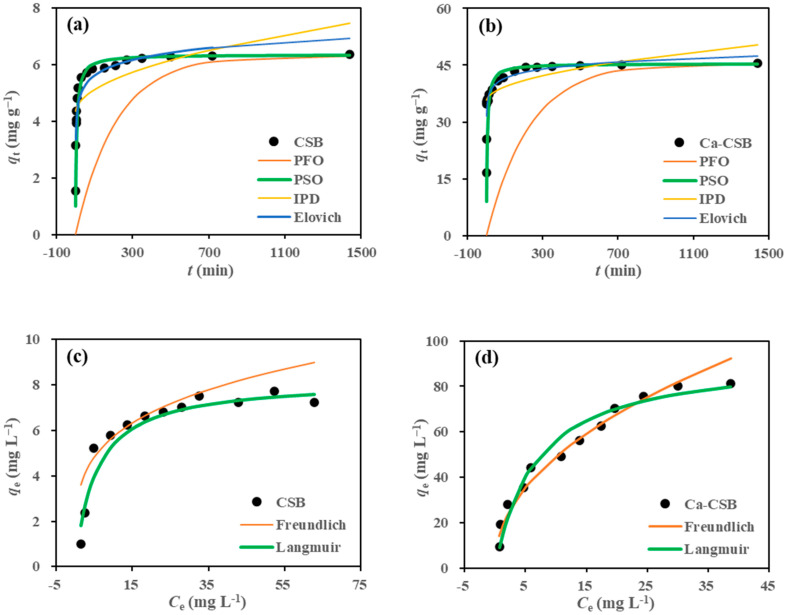
(**a**) Effect of time and fitting curves of the pseudo-first-order (PFO), pseudo-second-order (PSO), and the intra-particle diffusion (IPD) and Elovich kinetic models for tetracycline (TC) adsorption by corn straw biochar (CSB); (**b**) effect of time and fitting curves of the PFO, PSO, IPD, and Elovich models for TC adsorption by calcium hydroxide-modified CSB (Ca-CSB); (**c**) variation trend in the adsorption capacity *q*_e_ of CSB with TC equilibrium concentration *C*_e_ and Langmuir and Freundlich fitting curves on the adsorption of TC by CSB; and (**d**) variation trend in the adsorption capacity *q*_e_ of CSB with TC equilibrium concentration *C*_e_ and Langmuir and Freundlich fitting curves on the adsorption of TC by Ca-CSB (biochar dosage: 1.0 g L^−1^; TC concentration: 50 mg L^−1^ for kinetic; initial TC solution pH: 6.0; shaking time: 24 h for isotherm; 25 °C).

The adsorption kinetics of TC on CSB and Ca-CSB were determined using initial TC concentrations of 10 mg L^−1^ for CSB and 100 mg L^−1^ for Ca-CSB. The TC adsorption rate by both types of biochar was high within the first 30 min and then gradually decreased, reaching TC removal efficiencies of 87.3 and 84.9% for CSB and Ca-CSB, respectively, at an equilibrium time of 1440 min. Subsequently, the adsorption rate remained stable between 30 and 1440 min. The adsorption kinetics curves for TC are presented in Figure 3a,b.

Four adsorption kinetic models, namely, the PFO, PSO, IPD, and Elovich models, were selected to fit the experimental data; the derived parameters are summarised in Table 1. For both CSB and Ca-CSB, the PSO model provided the best fit for the TC adsorption behaviour, with an *R*^2^ value of 0.9999, indicating that chemical adsorption plays a dominant role in the adsorption of TC by both types of biochar [30].

The PFO and PSO models assume that the concentration difference in the adsorbate in the solid and liquid phases drives the adsorption process. The PFO model is more suitable for fast-rate adsorption processes, while the PSO model is more suitable for complex multistep adsorption processes [31]. The PSO model exhibited a significantly higher correlation coefficient (*R*^2^) than the PFO model, suggesting that TC adsorption by biochar involves a complex multistep process.

The Elovich kinetic model is commonly used to explain the chemisorption behaviour of highly heterogeneous adsorbents [32]. In this study, the correlation coefficients of the Elovich kinetic model in fitting CSB and Ca-CSB were 0.8391 and 0.7877, respectively, supporting the idea that chemical processes have multiple layers in TC adsorption. The initial adsorption rate coefficient (*α*) for Ca-CSB (23,184.4) was much larger than that for CSB (175.5), indicating a higher initial TC adsorption capacity for Ca-CSB. Additionally, the desorption constant (*β*) of the Elovich model was <2.0, suggesting that the TC adsorption process by biochar was stable with a low desorption rate, which can be considered negligible compared to the initial adsorption rate.

The Langmuir and Freundlich isothermal adsorption models were used to describe the adsorption capacity of TC on CSB and Ca-CSB at 25 °C (Figure 3c,d). The relatively high *R*^2^ values of these two models (0.7617–0.9857) indicated the diversity of TC adsorption mechanisms on the biochars. The Langmuir model provided a better fit to the experimental data for TC adsorption on both CSB and Ca-CSB, with *R*^2^ values of 0.9857 and 0.9757, respectively, compared to the Freundlich model (*R*^2^ values of 0.7617 and 0.9368, respectively; Table 1). The superior fitting result of the Langmuir equation suggests that TC adsorption on the biochars followed a non-linear isothermal adsorption process, primarily involving chemical adsorption and multiple forces. The maximum adsorption capacities (*q*_max_) calculated using the Langmuir equation were 8.22 mg g^−1^ for CSB and 93.46 mg g^−1^ for Ca-CSB, indicating that the maximum TC adsorption capacity on Ca-CSB was 10.4 times higher than that on CSB. In the Freundlich model, the *K*_F_ values for CSB and Ca-CSB were 1.57 and 16.64, respectively, indicating a significantly strong adsorption capacity for Ca-CSB. The *n*-values for CSB and Ca-CSB were 2.24 and 2.13, respectively, suggesting effective TC adsorption by both biochar types.

Previous studies have reported excellent TC adsorption performance of alkali-modified biochar. For example, the maximum TC adsorption capacity of KOH-modified biochar derived from rape straw [16] and peanut shells [33] reached 325.07 and 356.19 mg g^−1^, respectively. The maximum TC adsorption capacity of sodium hydroxide-modified biochar derived from spent coffee grounds was 113.64 mg g^−1^, which was 2.9 times higher than that of the pristine biochar [17]. However, in these studies, sodium hydroxide- or potassium hydroxide-modified biochars were prepared via a two-step pyrolysis at higher temperatures. For instance, the biomass of rape straw was first pyrolysed into the original biochar at 700 °C and then modified with potassium hydroxide at a pyrolysis temperature of 700 °C [16]. A similar preparation process was reported by Zhang et al., [33] for potassium hydroxide-modified biochar derived from peanut shells.

In this study, considering energy conservation, all modified biochars were pyrolysed at temperatures below 600 °C. Table 2 presents a comparison of the TC adsorption performance of the alkali-modified biochar samples obtained in this study and those reported in the literature. At pyrolysis temperatures below 600 °C, the TC adsorption capacity of Ca-CSB was higher than that of the other modified biochars derived from agricultural plants (except sugarcane bagasse; Table 2). This indicates that the calcium hydroxide-modified biochar prepared in this study achieved a high TC removal efficiency in wastewater streams with energy-saving benefits.

### 3.4. Adsorption Mechanism

To elucidate the adsorption mechanism of alkali modification on TC adsorption, CSB, Na-CSB, and Ca-CSB biochars were characterised. The specific surface areas and pore data are listed in Table 3. After modification with sodium hydroxide and calcium hydroxide, the specific surface area of CSB increased from 1.72 to 13.52 and 31.25 m^2^ g^−1^, respectively. A similar increasing trend was observed for the total pore volume. However, after alkali activation, the average pore size decreased from 23.97 to 11.33 nm for Na-CSB and 7.64 nm for Ca-CSB. Since the molecular size of TC is 1.23 × 0.84 × 0.67 nm^3^ [12], the decrease in pore size after alkali modification does not prevent TC from filling and diffusion. Instead, filling and diffusion increase with an increase in specific surface area and total pore volume. Therefore, both sodium hydroxide and calcium hydroxide modifications improved the TC adsorption capacity of CSB, with calcium hydroxide having a better modification effect than sodium hydroxide.

The C, N, O, and H concentrations and related atomic ratios of CSB, Na-CSBs, and Ca-CSBs are presented in Table 3. After modification with sodium hydroxide or calcium hydroxide, the C and N contents in CSB biochar decreased owing to the increase in Na and Ca, whereas the H and O contents increased owing to the introduction of OH [23]. The H/C, O/C, and (O+N)/C ratios in Na-CSB and Ca-CSB were higher than those in CSB, suggesting a reduction in the aromaticity of the modified biochar and an improvement in hydrophilicity and polarity [39], which are conducive to the adsorption of water-soluble TC.

As shown in Table 3 (EDX spectroscopy), the C content on the surface of Na-CSB and Ca-CSB decreased compared to that on CSB, while the O content increased. The surface of Na-CSB comprised 13.88% Na while that of Ca-CSB comprised 21.70% Ca, indicating successful surface modification. Furthermore, sodium hydroxide and calcium hydroxide reduced the Si content on the CSB surface by 50.7 and 36.1%, respectively, suggesting that both alkalis strongly solubilised Si on the CSB surface. Therefore, based on the elemental analysis and EDX results, both calcium hydroxide and sodium hydroxide improved the TC adsorption capacity of CSB by enhancing the polarity and hydrophilicity of the CSB surface. The increase in polarity and hydrophilicity caused by calcium hydroxide was higher than that caused by sodium hydroxide. Moreover, Ca on the surface of Ca-CSB formed a TC complex [35,40], explaining the better TC adsorption performance of Ca-CSB compared to that of Na-CSB.

**Figure 4:**
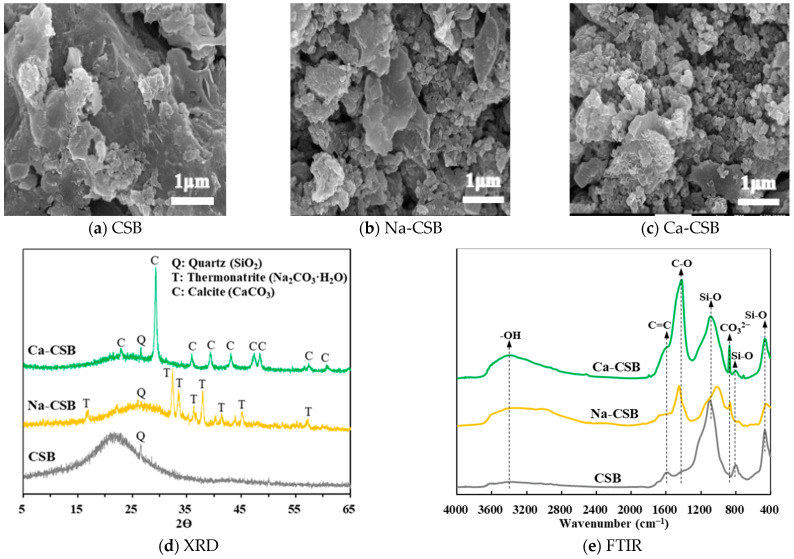
(**a**) Scanning electron microscopy (SEM) images of corn straw biochar (CSB), (**b**) sodium-hydroxide-modified corn straw biochar (Na-CSB), (**c**) calcium hydroxide-modified CSB (Ca-CSB), (**d**) X-ray powder diffraction (XRD) patterns, and (**e**) Fourier transform infrared (FTIR) spectra of CSB, Na-CSB, and Ca-CSB.

As shown in Figure 4a–c (SEM images), after modification with calcium hydroxide and sodium hydroxide, the surface of CSB became rough and fragmented, mainly due to a decrease in the size of macropores and an increase in the size of mesopores and micropores, increasing specific surface area and pore volume.

XRD was used to investigate the crystallinity of CSB, Na-CSB, and Ca-CSB (Figure 4d). The predominant peak of CSB was quartz (SiO_2_) at 26.6°. After modification with calcium hydroxide and sodium hydroxide, the intensities of the peaks decreased, indicating the dissolution of SiO_2_ under alkaline conditions, consistent with the EDX results. Notably, the peaks of Na-CSB were mainly associated with thermonatrite (Na_2_CO_3_·H_2_O) at 16.9°, 32.4°, 33.4°, 36.3°, 37.9°, 41.3°, 45.1°, and 57.3°, while the peaks of Ca-CSB were primarily assigned to calcite (CaCO_3_) at 22.9°, 29.3°, 35.9°, 39.4°, 43.1°, 47.4°, 48.4°, 57.5°, and 60.7°. The formation of these carbonate crystal peaks was primarily attributed to the oxidation of C to CO_2_, which further interacted with sodium hydroxide and calcium hydroxide to generate Na_2_CO_3_ and CaCO_3_, respectively [22,23]. CaCO_3_ can release Ca^2+^ when dissolved under acidic conditions [41], thereby forming complexes with TC, which partly explains the enhanced adsorption capacity of Ca-CSB for TC under acidic conditions [42].

As shown in Figure 4e (FTIR spectra), an extremely weak intensity of the functional group at 3386 cm^−1^ assigned to –OH was observed on the surface of CSB, while the intensity of the OH-group peak on the Na-CSB and Ca-CSB surfaces was significantly enhanced by the introduction of OH-groups in the base [16]. The OH-group peak can enhance TC adsorption through hydrogen bonding [23]. The intensity of the C=C peak on the surface of CSB at 1588 cm^−1^ was weakened or disappeared after calcium hydroxide and sodium hydroxide treatment, indicating a decrease in TC adsorption, mainly due to π–π interactions [18]. The C-O bond of CSB at 1418 cm^−1^ was significantly enhanced on the Ca-CSB and Na-CSB surfaces, with a blue shift to 1451 cm^−1^. The presence of a large number of C-O and OH groups of neutral TC^0^ on the Ca-CSB surface can significantly increase adsorption capacity [23,34].

There were three noticeable Si-O peaks located at 1102, 801, and 468 cm^−1^ on the CSB surface; on the Na-CSB and Ca-CSB surfaces, these peaks were weakened due to the destruction of Si-O bonds by calcium hydroxide and sodium hydroxide [34]. The intensity of these three peaks on the Na-CSB surface weakened more than that of the same peaks on the Ca-CSB surface, and the position of the peaks shifted, resulting in a weaker interaction between Si-O and TC on the Na-CSB surface than on the Ca-CSB surface. Thus, the TC adsorption capacity of Na-CSB was lower than that of Ca-CSB. CO_3_^2−^ peaks were observed on the Na-CSB and Ca-CSB surfaces with positions at 865 and 873 cm^−1^, respectively, which were absent in CSB, indicating the presence of several CO_3_^2−^ ions that facilitated an increase in the number of O-containing functional groups on the surface of Ca-CSB, improving the adsorption of TC and further increasing the gap between the adsorption capacities of Ca-CSB and Na-CSB.

**Figure 5:**
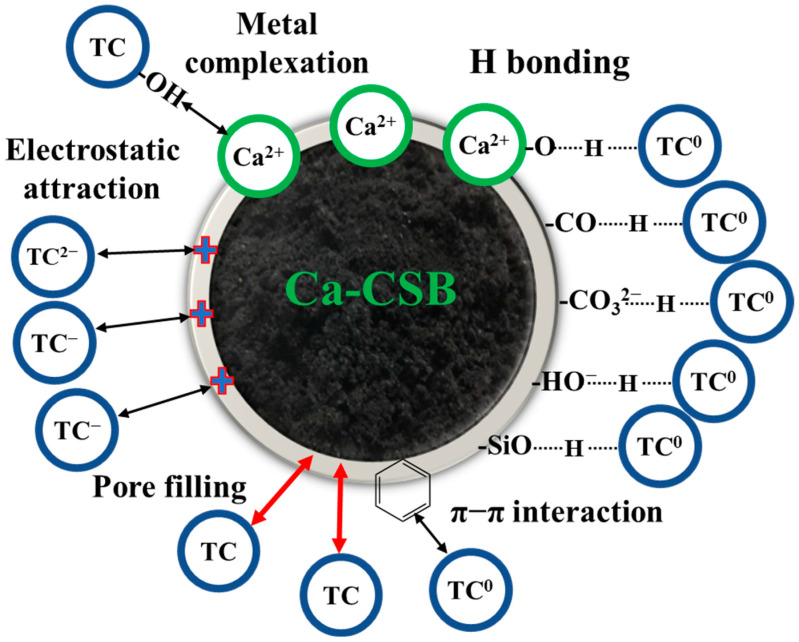
Mechanism underlying the adsorption of tetracycline (TC) on calcium hydroxide-modified corn straw biochar (Ca-CSB).

Therefore, compared to CSB and Na-CSB, the higher adsorption capacity of TC by Ca-CSB can be attributed to the following aspects: (1) at pH range between 7.3 and 9.7, electrostatic attraction occurs between the positively charged biochar surface and negatively charged TC surface; (2) at a lower pH, TC and the Ca^2+^ produced by the dissolution of CaCO_3_ on the surface of Ca-CSB form a complex; (3) several -OH, C-O, Si-O, and CO_3_^2−^ oxygen-containing functional groups on the surface interact with TC functional groups via hydrogen bonding; (4) π–π interactions between C=C bonds and TC occur; and (5) pore diffusion. The mechanism underlying TC adsorption on Ca-CSBs is summarised in Figure 5. In general, Ca-CSB is a green, affordable, and efficient TC adsorbent with great potential and prospect in the treatment of wastewater containing antibiotics.

## 4. Conclusions

Among the nine types of agricultural biomasses studied, calcium hydroxide exhibited the most effective modification effect on straw biomass. Ca-CSB demonstrated pH buffering capacity, maintaining the pH at 9.6, which was found to be the optimum pH for TC adsorption by Ca-CSB. Moreover, Ca-CSB showed a higher maximum adsorption capacity than the other biochars prepared at pyrolysis temperatures of 500 °C. The adsorption process followed the PSO kinetic model and Langmuir isotherm adsorption. The dominant mechanisms involved in TC adsorption by Ca-CSB were hydrogen bonding, oxygen-containing functional groups, Ca^2+^-metal complexation, and electrostatic attraction.

## Figures and Tables

**Table 1:** Kinetic and isotherm parameters for tetracycline (TC) adsorption by corn straw biochar (CSB) and calcium hydroxide-modified CSB (Ca-CSB).

Models	Equations	Parameters	CSB	Ca-CSB
PFO model	$q_{t}=q_{e}(1-e^{-k_{1}t})$	*k*_1_ (min^−1^)	0.3086	0.4754
		*q*_e_ (mg g^−1^)	5.90	42.20
		*R* ^2^	0.9116	0.8515
PSO model	$q_{t}=q_{e}-\frac{q_{e}}{1\;+\;k_{2}q_{e}t}$	*k*_2_ (g mg^−1^ min^−1^)	0.0287	0.0052
		*q*_e_ (mg g^−1^)	6.36	45.45
		*R* ^2^	0.9999	0.9999
IPD model	$q_{t}=k_{3}t^{\frac{1}{2}}+C$	*k*_3_ (g mg^−1^ min^−1/2^)	0.0897	0.5111
		*C* (mg g^−1^)	4.1482	32.8770
		*R* ^2^	0.5035	0.4840
Elovich model	$q_{t}=\frac{1}{\beta }\ln\left(\alpha \beta \right)+\frac{1}{\beta }\ln t$	*α* (mg g^−1^ min)	175.5	23,184.4
		*β* (g mg^−1^)	1.856	0.329
		*R* ^2^	0.8391	0.7877
Langmuir model	$q_{e}=\frac{q_{m}K_{L}C_{e}}{1\;+\;K_{L}C_{e}}$	*q*_m_ (mg g^−1^)	8.22	93.46
		*K*_L_ (L mg^−1^)	0.19	0.15
		*R* ^2^	0.9857	0.9757
Freundlich model	$q_{e}=K_{F}C_{e}^{1/n}$	*K*_F_ ((mg g^−1^) (L mg^−1^)^1/n^)	1.57	16.64
		n	2.24	2.13
		*R* ^2^	0.7617	0.9368

**Table 2:** Maximum tetracycline (TC) adsorption capacity of modified plant-derived biochar prepared at <600 °C.

Biochar Feedstock	Pyrolysis Temperature (°C)	Modified Reagent or Method	Maximum Adsorption Capacity (mg g^−1^)	Reference
Corn straw	500	Ca(OH)_2_	93.5	This study
Rice husk	500	KOH	58.8	Liu et al., 2012 [34]
Corn stover	500	CaCl_2_	12.5	Zhuo et al., 2022 [35]
Yellow pine wood	550	Ca(OH)_2_	20.9	Zeng and Kan, 2022 [23]
Pine sawdust Wheat straw	300–600	Air-limitation CO_2_-flow N_2_-flow	2.57–56.0 7.40–22.8	Xiang et al., 2022 [36]
Corn straw	500	K_2_FeO_4_ + ball milling	74.0	Qu et al., 2022 [15]
Tea waste	500 600	Hydrothermal + KHCO_3_ + FeCl_3_	59.4 86.2	Li et al., 2022 [37]
Sugarcane bagasse	600	FeCl_3_ + AlCl_3_ + ball milling	116.6	Tang et al., 2023 [38]

**Table 3:** Specific surface area, pore structure, and main elemental composition of corn straw biochar (CSB), sodium hydroxide-modified rice straw biochar (Na-CSB), and calcium hydroxide-modified CSB (Ca-CSB).

Properties	CSB	Na-CSB	Ca-CSB
S_BET_ (m^2^ g^−1^)	1.72	13.52	31.25
V_total_ (cm^3^ g^−1^)	0.01	0.04	0.06
D_ap_ (nm)	23.97	11.33	7.64
Ultimate analysis (wt%)			
C	35.74	29.74	20.17
H	1.86	2.00	2.08
O	17.92	21.73	19.65
N	0.57	0.10	0.48
H/C	0.62	0.81	1.24
O/C	0.38	0.55	0.73
(O+N)/C	0.39	0.55	0.75
EDX (wt%)			
C K	43.11	20.22	20.11
O K	25.42	48.87	46.09
Si K	17.28	8.52	11.04
Na K	—	13.88	—
Ca K	—	—	21.70
Other	14.19	8.51	1.06
O/C	0.44	1.81	1.72

## Data Availability

Data are contained within the article.
